# Interactions of Polyamines and Phytohormones in Plant Response to Abiotic Stress

**DOI:** 10.3390/plants12051159

**Published:** 2023-03-03

**Authors:** Natalia Napieraj, Małgorzata Janicka, Małgorzata Reda

**Affiliations:** Department of Plant Molecular Physiology, Faculty of Biological Sciences, University of Wrocław, Kanonia 6/8, 50-328 Wroclaw, Poland

**Keywords:** abiotic stress, polyamines, abscisic acid, brassinosteroids, ethylene, gibberellins, jasmonates, plant hormones

## Abstract

Numerous environmental conditions negatively affect plant production. Abiotic stresses, such as salinity, drought, temperature, and heavy metals, cause damage at the physiological, biochemical, and molecular level, and limit plant growth, development, and survival. Studies have indicated that small amine compounds, polyamines (PAs), play a key role in plant tolerance to various abiotic stresses. Pharmacological and molecular studies, as well as research using genetic and transgenic approaches, have revealed the favorable effects of PAs on growth, ion homeostasis, water maintenance, photosynthesis, reactive oxygen species (ROS) accumulation, and antioxidant systems in many plant species under abiotic stress. PAs display a multitrack action: regulating the expression of stress response genes and the activity of ion channels; improving the stability of membranes, DNA, and other biomolecules; and interacting with signaling molecules and plant hormones. In recent years the number of reports indicating crosstalk between PAs and phytohormones in plant response to abiotic stresses has increased. Interestingly, some plant hormones, previously known as plant growth regulators, can also participate in plant response to abiotic stresses. Therefore, the main goal of this review is to summarize the most significant results that represent the interactions between PAs and plant hormones, such as abscisic acid, brassinosteroids, ethylene, jasmonates, and gibberellins, in plants under abiotic stress. The future perspectives for research focusing on the crosstalk between PAs and plant hormones were also discussed.

## 1. Introduction

**Figure 1:**
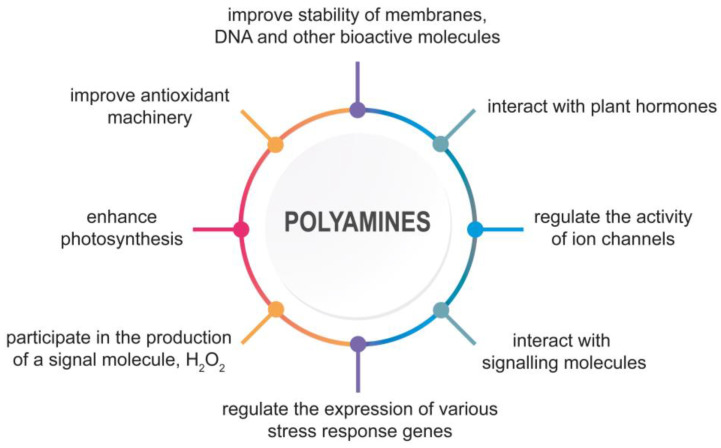
The multitrack action of PAs in plant response and tolerance to abiotic stresses.

Several environmental conditions can negatively affect plant production. Abiotic stresses, such as salt, drought, cold, heat, and heavy metal toxicity, cause plant damage at the physiological, biochemical and molecular level [1]. Salinity and drought are significant factors that threaten global agriculture [2,3,4]. Low temperatures are also considered to be a crucial harmful stress [5]. Moreover, elevated concentrations of heavy metals degrade soil health and, consequently, disrupt plant growth [6,7]. Abiotic stresses disrupt the water balance, membrane permeability, mineral nutrients uptake, and accumulation. Furthermore, photosynthesis, respiration, and protein synthesis are also limited. In plants, under various abiotic stresses, an imbalance between the production of reactive oxygen species (ROS) and their metabolism is observed. Oxidative stress negatively affects biomolecules and cellular organelles [8]. In response to unfavorable conditions, plants undergo physiological and metabolic reactions accompanied by modifications at the post-translational and post-transcriptional levels. Enhanced polyamines (PAs) accumulation is one of the most remarkable metabolic signs in plants subjected to abiotic stressors [5]. PAs are ubiquitous, polycationic, and aliphatic amines that play a significant role in physiological and developmental processes such as organogenesis, embryogenesis, cell division, floral development, reproductive organ development, root growth, leaf senescence, and fruit maturation [9,10]. PAs are also involved in plant response to biotic and abiotic stresses such as salinity, drought, heavy metals, and high- and low-temperature stresses [11,12,13,14]. Studies have shown that PAs display multi-track actions in plant response and stress tolerance. PAs improve the stability of membranes, DNA, and other bioactive molecules. They participate in chromatin condensation, maintenance of DNA structure, RNA processing, translation, and protein activation [15,16,17]. During abiotic stress, PAs modulate the expression of genes related to starch metabolism, photosynthesis, antioxidant machinery, and stress response [18,19,20,21]. PAs also play a significant role in the regulation of ion uptake and maintenance of ionic homeostasis [22]. For example, PAs directly inhibit fast- and slow-acting non-selective cation channels (NSCCs) in tonoplast with high affinity and reduce the efflux of toxic Na+ ions from vacuole to cytoplasm during salinity [23,24,25]. PAs can also activate Ca^2+^-ATPase localized in the plasma membrane and tonoplast, thereby regulating Ca^2+^ transport and preventing excessive Ca^2+^ level during prolonged exposure to stress [25,26,27]. PAs positively affect antioxidant machinery, improve osmolyte accumulation, enhance photosynthesis, and maintain nitrogen metabolism [13,28,29,30]. PAs interact with plant hormones, such as abscisic acid (ABA), ethylene (ETH), jasmonates (JAs), and gibberellins (GAs), and signaling molecules, such as γ-aminobutyric acid (GABA) and nitric oxide (NO), to enhance plant tolerance of abiotic stresses [31,32,33,34,35]. They are also involved in the production of a signal molecule, hydrogen peroxide (H_2_O_2_), due to PAs catabolism [36]. A consequence of PA-induced H_2_O_2_ generation may be the induction of stress-response genes or programmed cell death (PCD) [37,38] (Figure 1).

Plant hormones, commonly known as plant growth regulators, control growth and developmental processes that play a meaningful role in plant adaptation to unfavorable environments. They are divided into two groups: (I) positive growth regulators, such as auxins, cytokinins (CKs), GAs, and brassinosteroids (BRs); and (II) stress hormones, such as ABA, JAs, salicylic acid (SA), and ETH [39]. The additive effect of phytohormones on plant tolerance of abiotic stresses has been known for many years and has been extensively reviewed [40,41,42,43]. The literature has shown that plant hormones interact with PAs during plant growth and development (Table 1). Plant hormones regulate the expression of PAs metabolic genes [44,45,46]. On the other hand, PAs can regulate the biosynthesis, signal transduction, and transport of certain plant hormones [47,48,49]. Interestingly, some studies have revealed that plant hormones can participate in plant defense through interactions with PAs, inducing plant tolerance of abiotic stresses [39,50,51]. Although it has been known that PAs can interact with plant hormones, recent studies have gradually brought us closer to understanding the mechanisms of crosstalk between PAs and phytohormones during abiotic stress. Therefore, this paper aims to summarize the data on the relationship between PAs and hormones in plants subjected to various abiotic stresses. The potential direction for further studies related to this research area, which may lead to the discovery of detailed molecular mechanisms, is discussed.

## 2. PAs Metabolism

**Figure 2:**
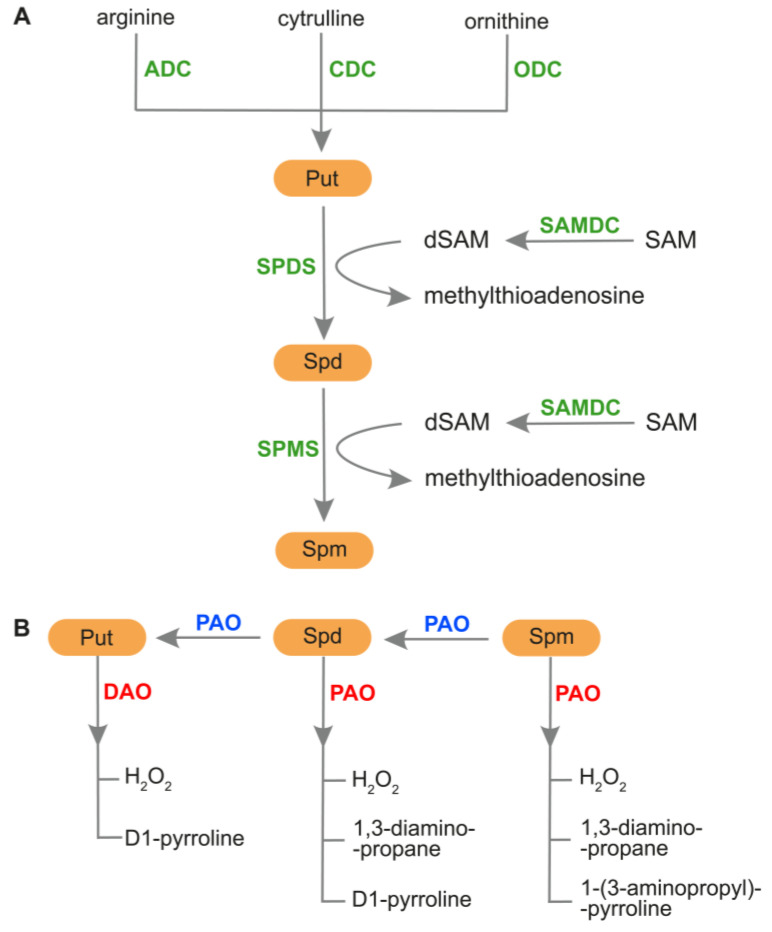
Metabolism of PAs in plants. (**A**) PAs biosynthesis. Production of Put can be catalyzed by ADC form arginine, by ODC from ornithine, or by CDC from citrulline. In the next step, SPDS is responsible for biosynthesis of Spd from Put, and SPMS participates in the production of Spm from Spd. During this process, dSAM is used as an aminopropyl donor to produce Spd and Spm. An enzyme, SAMDC takes part in the conversion of SAM to the form of dSAM. (**B**) PAs catabolism and back-conversion. DAO catalyzes Put oxidation during which D1-pyrroline and H_2_O_2_ are generated. PAO plays a role in Spd and Spm oxidation, producing H_2_O_2_, 1,3-diaminopropane and 1-pyrroline or 1-(3-aminopropyl)-pyrroline. PAO can also participate in the back-conversion of PAs, during which Spm is converted into Spd and next into Put. ADC, arginine decarboxylase; CDC, citrulline decarboxylase; DAO, diamine oxidase; dSAM, decarboxylated S-adenosylmethionine; ODC, ornithine decarboxylase; PAO, polyamine oxidase; Put, putrescine; SAM, S-adenosylmethionine; SAMDC, S-adenosylmethionine decarboxylase; Spd, spermidine; SPDS, spermidine synthase; Spm, spermine; SPMS, spermine synthase.

The most common PAs in the plant kingdom are diamine putrescine (Put), triamine spermidine (Spd) and tetraamine spermine (Spm) [12]. Other PAs identified in plants include agmatine (Agm), cadaverine (Cad), and an isomer of Spm, thermospermine (tSpm) [5,34]. The routes of PAs biosynthesis and catabolism, as well as PAs back-conversion, are shown in Figure 2.

The production of Put in plant tissues is catalyzed by arginine decarboxylase (ADC) or ornithine decarboxylase (ODC). However, some plants, such as *Arabidopsis thaliana* and many members of the *Brassicaceae* family, have lost their *ODC* genes [35,66]. Another pathway for Put production has been reported in sesame, in which Put biosynthesis occurs from citrulline (Cit) in a reaction catalyzed by citrulline decarboxylase (CDC) [66]. After Put production, S-adenosyl-L-methionine decarboxylase (SAMDC) introduces S-adenosyl-L-methionine (SAM) into the pathway. Then, the decarboxylated form of SAM (dSAM) is used as an aminopropyl donor in the conversion of Put to Spd and then Spd to Spm. The reaction is catalyzed by Spd synthase (SPDS) and Spm synthase (SPMS), respectively [66]. Two enzymes, diamine oxidase (DAO; also known as CuAO) and polyamine oxidase (PAO), are involved in PAs catabolism. DAO catalyzes Put oxidation in the apoplast, resulting in the production of D1-pyrroline and H_2_O_2_. PAO participates in Spd and Spm oxidation in apoplast and peroxisomes, generating H_2_O_2_, 1,3-diaminopropane and 1-pyrroline or 1-(3-aminopropyl)-pyrroline [64,67,68,69,70]. The product of PAs catabolism, H_2_O_2_, plays a significant role in plant development and response to environmental stresses; however, overaccumulation of this molecule negatively affects plants [14,51,69]. It is worth noting that other products of PA catabolism are acrolein and GABA [14]. Some PAOs catalyze the back-conversion of higher PAs. During this process, Spm is converted into Spd and subsequently into Put [71].

## 3. Interaction of PAs and Plant Hormones under Abiotic Stress

The literature has indicated that PAs play a significant role in plant tolerance of abiotic stresses [5,64]. Furthermore, many studies have revealed that plant hormones are important elements in plant responses to adverse environmental conditions [41,70]. These two groups of molecules appear to be significant in the signal transduction pathway that is activated in plants in response to abiotic stress. Determining the interactions between PAs and plant hormones is crucial. Therefore, reports indicating crosstalk between PAs and phytohormones and their significance in improving plant tolerance to abiotic stresses have been summarized (Table 2).

### 3.1. PAs-Plant Hormones Crosstalk under Drought Stress

Drought is considered to be one of the most threatening abiotic stresses in plants. It has a negative effect on plant growth and crop productivity [39]. Drought leads to the loss of cell turgor, water and mineral disruption, and photosynthesis limitation [3,83]. A negative effect of drought is osmotic stress, which induces oxidative stress. Excessive accumulation of ROS can disrupt the functioning of cell membranes and induce lipid peroxidation and DNA modifications. This leads to metabolic and structural dysfunction and PCD [13,79]. 

**Figure 3:**
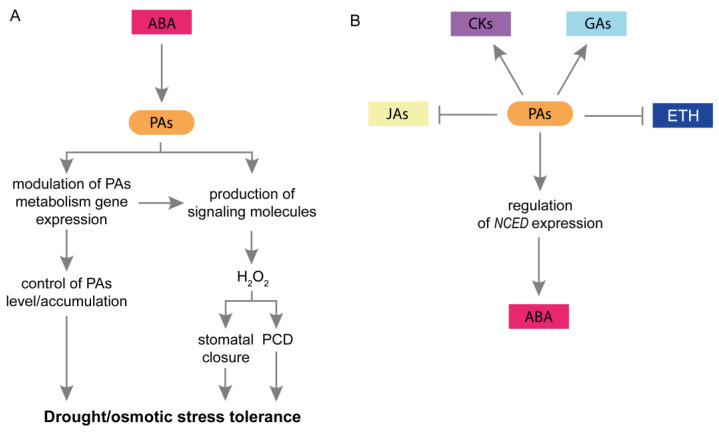
Interaction between PAs and plant hormones in response to drought and osmotic stress. (**A**) The effect of ABA on PAs’ mode of action. ABA modulates the expression of PAs metabolism genes and controls PAs content, thereby inducing plant tolerance to drought and osmotic stress. Furthermore, due to its impact on PAs catabolism, ABA regulates the production of H_2_O_2_, which may induce stomatal closure or PCD. (**B**) A schematic representation of possible PA-plant hormone interactions during plant responses to drought and osmotic stress. During drought/osmotic stress, PAs regulate *NCED* expression and ABA content. They also increase CKs and GAs content and reduce ETH and JAs content, probably inducing plant tolerance to stress. ABA, abscisic acid; CKs, cytokinins; ETH, ethylene; GAs, gibberellins; H_2_O_2_, hydrogen peroxide; JAs, jasmonates; NCED, 9-cis-epoxycarotenoid dioxygenase; PAs, polyamines; PCD, programmed cell death.

The essential mechanisms of plant response to drought include the participation of ABA. This phytohormone is involved in seed dormancy, embryo morphogenesis, and fat and stored protein production [43]. During these processes, ABA interacts with multiple plant hormones, signaling molecules, and secondary metabolites such as benzoxazinoids [72,94,95,96]. During drought, ABA stimulates stomatal closure to prevent excessive water loss from leaves, modulates the expression of multiple genes involved in the plant response to stress, and modifies the biosynthesis of osmoprotectants [21]. Studies have revealed that exogenous ABA increased plant tolerance to drought by improving cell membrane stability, stimulation of antioxidant enzymes, and reduction of ROS content [43]. Previous studies have provided evidence for crosstalk between PAs and ABA (Figure 3A). Exogenous PAs induce stomatal closure in *Vicia faba* guard cells [73]. On the other hand, ABA has been reported to stimulate the activity of CuAO and NADPH oxidase, leading to enhanced H_2_O_2_ production in guard cells. Consequently, H_2_O_2_ induces Ca^2+^ influx into guard cells, promoting stomatal closure [73]. Comprehensive studies of *Arabidopsis thaliana*, have also revealed the role of PAs in regulating stomatal movement. It has been suggested that Spm may contribute to increasing cytoplasmic Ca^2+^ concentration by activating specific ion channels, especially Ca^2+^-permeable channels, which inactivate the K^+^ inward rectifier at the plasma membrane. This could stimulate stomatal closure [11]. Furthermore, the application of exogenous PAs increase NO and ROS levels in guard cells. PAs induce ROS production via NADPH oxidase and amine oxidase (AO) [75]. It has been suggested that the action of PAs and ABA is linked during stomatal closure due to the involvement of the same key components: ROS and NO [75,97]. The relationship between PAs and ABA in the regulation of stomatal movement in *Arabidopsis* is supported by Fincato et al. [74]. The ABA-induced expression of *AtPAO2* has also been reported in guard cells. Moreover, Fraudentali et al. [98] described that AtCuAOδ participates in ABA-induced stomatal closure. 

It has also been proposed that PAs are involved in plant tolerance and acclimation to drought by modulating ABA biosynthesis. Appropriate ABA content is important for plant stress tolerance. Excessive ABA accumulation may lead to reduced plant tolerance to drought [83]. Marcińska et al. [82] examined the effect of exogenous PAs on stress tolerance in two wheat genotypes exposed to osmotic stress. In drought-resistant plants, exogenous PAs (especially Spd) reduce the ABA content during osmotic stress. This may indicate the role of PAs in alleviating the adverse effects of stress and the negative physiological effects of drought. In contrast, in drought-sensitive wheat seedlings, the application of PAs (especially Spm) led to further increases in the ABA content. The authors suggested that an increase in ABA levels reflects a high intensity of drought response. Plants attempt to avoid the lethal effects of stress and reduce water loss through stomata [82]. PAs may regulate ABA content at the genetic level by modulation of genes encoding ABA biosynthesis enzymes. ABA is produced from C40 carotenoids in three steps catalyzed by zeaxanthin epoxidase, 9-cis-epoxycarotenoid dioxygenase (NCED), and abscisic aldehyde oxidase (AAO) [99,100,101]. Put strongly enhanced the expression of *NCED* in wheat leaves under osmotic and drought conditions [78,79]. 

Several studies have described the relationship between ABA and PAs metabolism. Exogenous ABA up-regulates the expression of several genes involved in PA production in *Arabidopsis* [45]. PAO activity and expression of *PAO* were induced by exogenous ABA [52,54]. A complex expression profile of seven *PAO* genes was also presented in tomato plants treated with exogenous ABA [44]. During drought, ABA may induce synchronized effects on the anabolism/catabolism of PA to increase the cellular ROS load for signaling downstream stress defense events. In *Vitis vinifera*, ABA stimulates the activities of enzymes involved in PA biosynthesis and, at least partially, induces PA accumulation and their exodus into the apoplast. At the same time, ABA is an upstream signal for the induction of PAs catabolism. Accumulated PAs begin to oxidize primarily in the apoplast. A product of PAs oxidation, H_2_O_2_, may act as a secondary messenger in the signaling pathway, inducing stomatal closure and/or PCD [102,103]. Furthermore, ABA has been shown to modulate the expression of PAs production and catabolism genes in response to drought [81,104]. 

Higher ABA and PAs contents were observed under drought conditions in the transgenic *Arabidopsis* line overexpressing *OsHSFA3*, which encodes one of the heat-shock factors associated with improved tolerance to drought and high temperature [79]. This was related to the higher expression of the PAs biosynthesis genes (*ADC1*, *ADC2*, *SPDS1*, and *SPMS*). Furthermore, ABA-responsive elements (ABREs) have been reported in the promoter regions of these PAs biosynthetic genes, suggesting a role in the regulation of PA production [76,77]. Moreover, in the *Arabidopsis* exposed to drought, the expression of ABA-inducible and drought-responsive genes (*RD29A* and *RD22*) increased and showed similar kinetics as the expression of PAs biosynthesis genes (*ADC1*, *ADC2*, *SPDS1*, *SPDS2*, *SPMS*, and *SAMDC1*). This supports the role of ABA signaling in the regulation of PAs production at the transcriptional level [68]. In silico analysis by Basu et al. [78] revealed the presence of several putative cis-acting elements, such as ABRE, Low-Temperature Responsive Element (LTRE), MYB, and W-box, in the promoter region of rice *SAMDC*. These elements are strongly associated with environmental factors such as drought, cold, and ABA signaling [78]. 

Several studies have suggested a relationship between PAs and other phytohormones in plant response to drought (Figure 3B). For example, the application of exogenous PAs modulated GA and SA content in some plant species under drought conditions [39,83,85,105], probably increasing plant tolerance to stress. However, the physiological implications of these changes require further investigation. BRs are steroidal plant hormones that regulate plant growth and productivity. The exogenous application of BRs has revealed their role in plant tolerance to low and high temperatures, heavy metals, drought, salinity, and waterlogging [13,106]. Talaat et al. [13] reported that the co-application of 24-epibrassinolide (EBL), one of BRs forms, and Spm improved plant drought tolerance and reduced the accumulation of ROS by enhancing their scavenging through the elevation of antioxidant machinery. In this case, further studies are necessary to reveal mechanisms between PAs and BRs in plant response to this stress. 

JAs are other plant growth regulators involved in plant responses to stress through crosstalk with PAs. The JAs family includes methyl jasmonate (MeJA), jasmonic acid (JA), and jasmonyl-isoleucine [107]. JAs regulate the expression of genes responsible for tolerance, interact with other phytohormones, and modulate proteome profiles. They can function as stress modulators by suppressing or enhancing plant stress responses [108]. Peremarti et al. [84] showed that in rice MeJA negatively regulated *OsAdc*, *OsSamdc*, and *OsSpds* gene expression. According to the obtained results, it was proposed that drought-induced Put accumulation occurs until the threshold for conversion to higher PAs is reached. At the same time, activation of JAs signaling is important for the enhancement of Put conjugation, reduction of free Put content, and withdrawal of plants from the threshold [84]. On the other hand, PAs decrease JAs accumulation in plants exposed to drought [43]. It is well known that the application of JAs retards plant growth, and activation of JAs signaling causes repression of photosynthesis and photosynthesis-related gene expression [109,110]. Therefore, it is suggested that PA-mediated reduction of JAs content may play a vital role in plant tolerance to stress [39].

More advanced studies have presented the crosstalk between PAs and ETH at several levels. ETH is a gaseous plant hormone that modulates a broad array of plant responses such as cell expansion, seed germination, flowering, fruit ripening, and senescence. It also affects growth parameters, osmolytes and pigment content, photosynthesis, and oxidative stress in plants subjected to different abiotic stresses [111]. It is suggested that ETH participates in the regulation of stomatal movement. However, the detailed role of this plant hormone in plant responses to drought has not been well established [43]. Interestingly, PAs and ETH are connected in their biosynthetic pathways. They have the same precursor, SAM, produced from L-methionine and ATP by S-adenosyl-L-methionine synthetase (SAMS) [33]. Hence, it has been suggested that PA and ETH compete for SAM production [31]. Recent studies have demonstrated that PAs regulate ETH content in some plant species. Grzesiak et al. [112] suggested that higher PAs content led to a reduction of ETH biosynthesis in PEG-treated wheat seedlings. Liu et al. [83] showed that the effect on ETH content might depend on the PAs’ type and lead to different results. A reduction in the ETH evolution rate was observed in the grain-filling of wheat under drought conditions. Application of Spd and Spm during drought resulted in further reduction of ETH evolution rate, whereas CKs and ABA content increased, thereby promoting wheat grain filling under drought conditions. Interestingly, an adverse effect was observed for exogenous Put, which increased ETH evolution rate and led to excessive ABA accumulation and inhibition of wheat grain filling [83]. Furthermore, Huang et al. [3] demonstrated that the activity of ribulose 1,5-bisphosphate carboxylase (RuBPC) was reduced in plants in response to drought. Aminooxyacetic acid (AOAA, an inhibitor of ethylene synthesis) may enhance RuBPC activity by modulating PAs levels and decreasing the damage caused by ETH release [3]. Additionally, a close relationship between PAs, ETH, and ROS was observed in the leaves of *Glycyrrhiza inflata* seedlings under osmotic stress. In severely damaged leaves, ROS production was modulated by PAs and ETH. Inhibition of ETH generation decreased ROS production, whereas a reduction in PAs content promoted ROS generation. Conversely, the exogenous application of H_2_O_2_ promoted ETH production and reduced PAs content [31].

### 3.2. PAs-Phytohormones Cross Talk under Salt Stress

Salinity is another factor that can reduce agricultural productivity [85]. According to estimates, salinity affects more than 800 million hectares of arable land worldwide. Salt stress reduces plant growth and disrupts photosynthesis and respiration [85]. Salinity causes the overproduction and accumulation of ROS in plants, which leads to salinity-induced osmotic stress [113,114].

Among all plant hormones, crosstalk between PAs and ETH during salt stress seems to be the most established. It has been suggested that plant tolerance to salinity may be related to ETH biosynthesis and signal transduction pathways. Exogenous application of ETH increased plant tolerance to salinity due to the increased expression of genes involved in ROS scavenging [115]. Furthermore, ETH may help regulate Na^+^ and K^+^ concentrations in xylem tissues under salt stress [111]. In contrast, the application of ETH biosynthesis inhibitors increases plant sensitivity to salt stress [115]. PAs can play a role in modulating ETH production during salinity. Quinet et al. [67] demonstrated that the application of exogenous Put enhanced ETH synthesis in the salt-resistant cultivar exposed to salinity [67]. Zapata et al. [115] noticed that under salinity, the ETH, Spd, and Spm contents increased in plant species with different salt sensitivity. PAs and ETH may compete for SAM production. However, the results support that no competence between them occurred, and the SAM pool is high enough to support both PAs and ETH biosynthesis during salt stress [31,115]. Because PAs catabolism is important in plant responses to stress, the crosstalk between PAs catabolism and ETH signaling has been investigated. In transgenic tobacco plants displaying reduced PAO activity, the transcript levels of genes encoding 1-amino-cyclopropane-carboxylate synthase (ACS) and 1-amino-cyclopropane-carboxylate oxidase (ACO) were higher than those in plants displaying enhanced PAO activity [4]. ACS converts SAM to 1-aminocyclopropane-1-carboxylic acid (ACC), whereas ACO converts ACC to ETH [116]. To provide further insight into the link between PAs, ETH, and H_2_O_2_, Freitas et al. [70] proposed a detailed model of salinity-induced expression and activity of ACO, leading to enhanced ETH biosynthesis in the salt-tolerant maize genotype. Consequently, ETH induces H_2_O_2_ production due to enhanced *DAO* expression and activity. H_2_O_2_ plays a dual role. It acts as a signaling molecule for PAs synthesis during the first stage of stress. In the second stage of stress, H_2_O_2_ inhibits “stress” ETH production and activates PAs biosynthesis by up-regulating *ADC* expression and activity. A recent study provided by Takács et al. [46] demonstrated that ETH-mediated signaling associated with NR receptors played a key role in PA metabolism in tomato fruits at the mature stage during salt stress conditions. In this study, the tomato ripening mutant *Never ripe* (*Nr*), which has a mutation in the ETH-binding domain of the ETH receptor NR, was used [117]. Among the three PAs tested, salt stress enhanced only Spm content in WT fruits. However, this change was not observed in *Nr* fruits exposed to salt stress. Moreover, during salinity stress, the activities of DAO and PAO were strongly stimulated in *Nr* fruits, contributing to higher oxidative stress [46]. 

BRs positively influence plant tolerance to salt stress. In *Arabidopsis*, enhanced BR signaling activity increases salt stress tolerance. In contrast, the BR-defective mutants exhibit hypersensitivity to salinity. Moreover, BRs may control water loss by reducing stomatal density in plants exposed to high salt stress conditions [40]. Co-application of BRs and PAs has a beneficial effect on plants exposed to salt stress [106,118]. Exogenous application of BRs enhances plant tolerance to salinity by modulating PAs homeostasis and distribution in plants [59,86]. Liu et al. [14] described a more advanced model of PAs and BRs interactions during plant response to low and high salt salinity conditions. EBL promotes tolerance of canola to high salt salinity, but not to low salt stress. The dual effect of EBL on plant responses to low and high salinity is related to H_2_O_2_ accumulation, which is regulated by PAs metabolism (especially Put oxidation). Under high-salt stress, EBL reduced the accumulation of H_2_O_2_ and DAO activity. Interestingly, EBL modulated these parameters in the opposite manner in seedlings exposed to low-salt stress [14].

**Figure 4:**
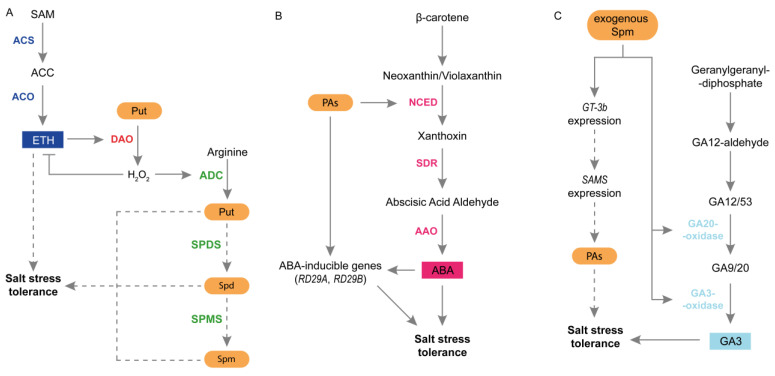
Interactions between PAs and plant hormones in plants subjected to salt stress. (**A**) Crosstalk between PAs and ETH during salinity stress in maize. In maize exposed to salt stress, the expression of *ACS* and *ACO* increased, leading to a higher ETH content. As a result, ETH stimulates *DAO* expression and activity, which oxidizes Put to H_2_O_2_. Next, H_2_O_2_ plays a dual role. It reduces ETH production and prevents excessive ETH content, which may have an unfavorable effect on plants. Moreover, H_2_O_2_ stimulates PA production by upregulating *ADC* expression and ACD activity. (**B**) Interactions between PAs and ABA in plant response to salt stress. PAs regulate the expression of *NCED* and ABA content. PAs also regulate the expression of other ABA-inducible genes, such as *RD29A* and *RD29B*, thereby inducing plant tolerance to salt stress. (**C**) PAs and GAs relationships during plant response to salt stress. Exogenous Spd stimulates the activity of GA20 oxidase and GA3 oxidase and up-regulates the expression of some genes involved in GA biosynthesis. Under salt-stress conditions, exogenous Spd enhances GA3 content and stimulates expression of the GT-3b transcription factor. Furthermore, exogenous Spd may promote the expression of *SAMS* via GT-3b and PAs production, thereby increasing plant tolerance to salt stress. AAO, abscisic aldehyde oxidase; ACC, 1-aminocyclopropane-1-carboxylic acid; ACO, 1-amino-cyclopropane-carboxylate oxidase; ACS, 1-amino-cyclopropane-carboxylate synthase; ADC, arginine decarboxylase; DAO, diamine oxidase; ETH, ethylene; GA, gibberellin; H_2_O_2_, hydrogen peroxide; NCED, 9-cis-epoxycarotenoid dioxygenase; PAs, polyamines; Put, putrescine; SAM, S-adenosyl-L-metionine; SAMS, S-adenosyl-L-metionine synthase; SDR, short-chain alcohol dehydrogenase/reductase; Spd, spermidine; SPDS, spermidine synthase; Spm, spermine; SPMS, spermine synthase.

A high endogenous ABA content is important for increasing plant tolerance to salt stress. ABA induces stomatal closure and accumulation of numerous proteins and osmoprotectants [40]. In the *atpao5-3* mutant, salt stress increased ABA level, up-regulated *NCED* expression and enhanced ABA-inducible *RD29B* expression [64]. On the other hand, the expression of *NCED3* was reduced in Spm-deficient *spms* mutant and double *acl5/spms* mutant under salt stress conditions. In addition, in transgenic *Arabidopsis* lines overexpressing *SAMDC1*, two of the five ABA-induced genes (*NCED* and *RD29A*) were up-regulated by salinity [81] (Figure 4B). 

GAs can also interact with PAs during plant response to salt stress. GAs are tetracyclic diterpenoid carboxylic acids that participate in processes related to plant growth and development [119]. GAs positively regulate germination, leaf expansion, and stem elongation. They also initiate flowering, trichome formation, and reproductive development [1]. Salt stress negatively affects endogenous GAs content in plants. This was accompanied by a higher accumulation of DELLA proteins, which are major GA-negative regulators. Interestingly, studies have shown that GA-deficient biosynthetic or signaling mutants exhibit tolerance to severe salt stress. In contrast, the quadruple DELLA loss-of-function mutant was less tolerant to salt [40]. Recent studies have shown that exogenous GAs improve plant tolerance to salinity. Positive effects of Spd and GA3 on priming-induced physiological and biochemical changes have been reported in salt-tolerant and salt-sensitive rice cultivars [2]. In cucumbers, exogenous Spd up-regulated the expression of genes related to GA biosynthesis and increased the activity of GA3-oxidase and GA20-oxidase. Moreover, in salt-stressed plants, exogenous Spd stimulated the accumulation of GA3 and enhanced the expression of the GT-3b transcription factor, a stress-related protein usually induced by salt stress. Spd-induced salt tolerance was reduced in plants treated with GA biosynthesis inhibitor. This suggests that GA plays a role in Spd-induced salt tolerance in cucumber [33] (Figure 4C). In the same species, the application of Spd to salt-treated plants reduced JA content [87]. The role of PAs in the regulation of JA content has also been demonstrated in *Arabidopsis* seedlings exposed to salinity. Exposure of the *atpao5-3* mutant to salt stress resulted in strongly enhanced JA accumulation [64]. 

The relationship between CKs and PAs in plant responses to salt stress has not been well described. CKs such as zeatin (Z) and zeatin ribose (ZR) are involved in plant cell division and growth, morphogenesis, chloroplast biogenesis, nutrient absorption and balance, and vascular differentiation. They also participate in seed germination and delay senescence in plants [39,51,83]. CKs play an essential role in plant responses to nutrient, drought, salinity, and temperature stresses [120]. Simultaneous application of kinetin (KN) and PAs improves tolerance to salt stress in some plant species, such as *Vigna sinensis*, wheat, and *Luffa acutangular* [50,51,88]. The positive influence of KN and Spm on photosynthesis parameters has been extensively described in wheat exposed to salt stress. Grain priming with KN, Spm, or KN + Spm alleviated the negative effects of stress by stimulating leaf area expansion, pigment production, photosynthetic activity, and improving chloroplasts ultrastructure [50]. Additionally, Kapoor and Hasanuzzaman [51] reported that exogenous KN and Put synergistically mitigate salt stress in sponge gourd (*Luffa acutangula* L.). Simultaneous application of KN and Put to salt-stressed seedlings resulted in a significant enhancement of growth, photosynthetic pigment content, and osmolyte accumulation. Moreover, the co-application of KN and Put up-regulated the antioxidant enzymes activity and increased the content of the non-enzymatic components of the antioxidant defense machinery. Simultaneous KN and Put treatment was more effective than individual application of KN or Put, suggesting the presence of regulatory crosstalk mechanisms between KN and Put [51]. However, the more advanced relationship between PAs and CKs remains unknown. 

### 3.3. PAs-Phytohormones Cross Talk under Heavy Metal Stress

Micronutrients such as copper (Cu), iron (Fe), manganese (Mn), nickel (Ni), and zinc (Zn) are heavy metals. These elements are significant in plants, but high concentrations of them have adverse effects [121]. In addition, other heavy metals, such as cadmium (Cd) and lead (Pb), present in the soil can cause stress conditions in plants. Uncontrolled accumulation of heavy metals in the soil has a negative impact on agriculture. Heavy metals disrupt plant growth, senescence, photosynthetic machinery efficiency, and enzymatic activity [89,90]. 

**Figure 5:**
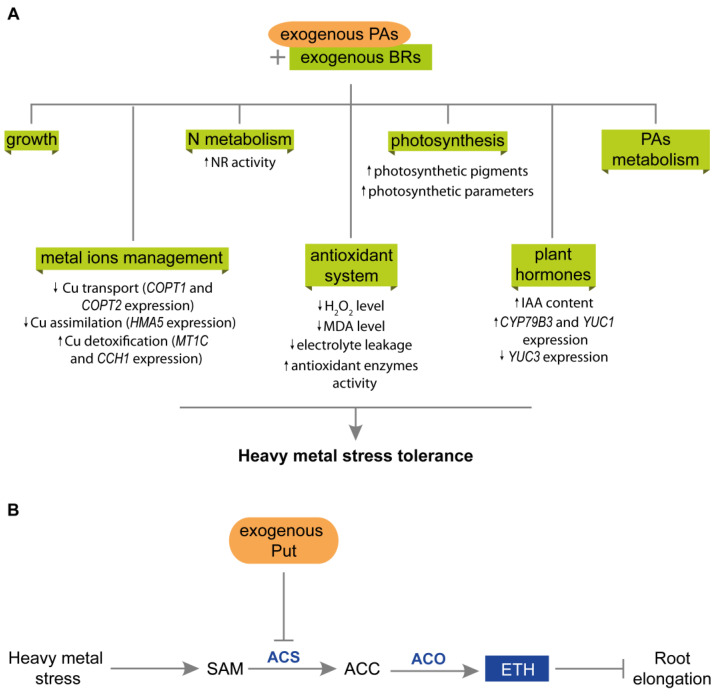
Interactions between PAs and plant hormones during plant responses to heavy metal stress. (**A**) Synergistic action of exogenous PAs and BRs in plants exposed to heavy-metal stress. PAs and BRs possibly cooperate at several levels, improving growth parameters, N metabolism, photosynthesis efficiency, and regulating PAs metabolism. They also manage metal ions uptake, distribution, and detoxification and enhance antioxidant machinery, thereby reducing the damage caused by ROS. The co-action of PAs and BRs leads to the enhanced expression of IAA metabolic genes and higher IAA content, probably stimulating plant growth and tolerance to stress. (**B**) Interactions between PAs and ETH during root elongation in plants exposed to heavy-metal stress. During heavy metal stress, ETH production increases, leading to inhibition of root elongation in plants. However, exogenous Put reduces ACS activity and ETH production; therefore, the inhibition of root elongation is declined. ACC, 1-aminocyclopropane-1-carboxylic acid; ACO, 1-amino-cyclopropane-carboxylate oxidase; ACS, 1-amino-cyclopropane-carboxylate synthase; BRs, brassinosteroids; ETH, ethylene; IAA, indole acetic acid; PAs, polyamines SAM, S-adenosyl-L-metionine.

Some reports suggest that PAs may interact with phytohormones, especially BRs and ETH, during plant responses to heavy metal stress [6,28] (Figure 5). However, the detailed mechanism of this cooperation has not yet been elucidated. The application of BRs and PAs enhanced the tolerance of some plant species to stress caused by Cr (VI) [28,56], Cu (II) [89,122], Mn [121], and Pb [90] (Figure 5A). Simultaneous BRs and PAs treatment improved growth parameters, reduced malondialdehyde (MDA) and H_2_O_2_ levels, and enhanced the antioxidant system [28,90,121,122]. To prevent heavy metal ion toxicity, plants have developed strategies to manage ion uptake, translocation, and distribution, involving BRs and PAs [42,122]. In plants exposed to Cu (II) stress, exogenous Spd and BRs down-regulated the expression of *COPT1* and *COPT2*, which encode the plasma membrane transporters responsible for Cu uptake into the cells. Furthermore, the co-application of Spd and BRs reduced the expression of the *HMA5* gene encoding heavy metal ATPases (HMAs), which play an important role in the transmembrane transport of Cu. Finally, the application of Spd and BRs up-regulated the expression of *MT1C* and *CCH1*, which encode the proteins involved in Cu detoxification [122]. Heavy metal sequestration is also a crucial process in plant responses to heavy metal stress. Biomolecules such as GSH and phytochelatins can act as metal-chelating agents [42]. Co-application of BRs and PAs increased phytochelatin and GSH content, as well as photosynthetic pigment content [28,122]. Moreover, EBL changed PAs accumulation in mustard under Cu (II) stress. The authors explained that the higher PA content (Spm, Spd, Put, and Cad) in the leaves of mustard exposed to Cu helped the plants mitigate this stress [89]. 

During heavy metal exposure, ETH production increases in some plant species [6]. ETH signaling is involved in the response of plants to oxidative stress induced by heavy metals. The exogenous application of ETH or ethephone mitigated the negative effects of heavy metal stress due to glutathione accumulation. Additionally, overexpression of *ERF* genes lead to increased expression of glutathione synthesis genes and increased Cd tolerance [42,111]. One of the adverse effects of plant exposure to Al toxicity is inhibition of root elongation. PAs and ETH are involved in this process [6] (Figure 5B). In wheat cultivars exposed to Al toxicity, Put supplementation alleviates the negative effects of stress. Further analyses revealed that ACS- and ACO-mediated Al-induced ETH production may play a role in Al-induced inhibition of root elongation. Application of Put decreases ACS activity and ETH production and reduces the inhibition of root elongation in plants exposed to Al toxicity [6]. Kohli et al. [90] noted the enhancement in Spd level in roots of mustard treated with EBL + SA and exposed to Pb stress. The positive effect of Spd on plant tolerance may be related to its antioxidant properties, which protect DNA from oxidative damage [90]. Shah et al. [7] described the positive correlation between PAs and ETH in cucumber seedlings supplemented with 2-hydroxymelatonin (2-OHMT) and exposed to Cd stress. In stressed seedlings, the application of 2-OHMT reduced PAO activity, leading to higher Put, Spd, and Spm content. The application of 2-OHMT also enhanced the expression of a gene encoding the ETH receptor (CS-ERS) during stress conditions [7]. 

### 3.4. PAs-Phytohormones Cross Talk under Low Temperature Stress

Low temperatures affect many physiological processes such as photosynthesis, nutrient absorption and metabolism, and can cause metabolic disruption and structural damage [5,35,123].

PAs probably maintain the proper ABA content in plants exposed to low-temperature stress. Higher ABA content enhanced tolerance of chilling by inducing stomatal closure and promoting the expression of cold-induced genes [91]. In an *Arabidopsis* subjected to low temperatures, a rapid increase in Put level is necessary for ABA accumulation [12]. Furthermore, Put enhances *NCED* expression in stressed plants [12]. In tomato plants exposed to chilling, exogenous Put increased ABA content and triggered the expression of *LeNCED1*. This indicates that ABA is involved in Put-induced tolerance to chilling stress [35]. Furthermore, *ADC2* contains an ABRE transcription factor in its’ promoter. Under low temperature stress, ABRE is responsible for the enhanced expression of genes mediated by ABA [77,124]. Li et al. [123] confirmed the role of *CmADC* as a positive regulator of melon-seedling cold tolerance and found two transcription factors, ABRE-binding factor 1 (CmABF1), and C-repeat binding factor 4 (CmCBF4), that directly target *CmADC* to enhance its expression. 

The crosstalk between PAs and JAs is also important for plant tolerance to low-temperature stress. In the exocarp of zucchini squash fruit (*Cucurbita pepo* L.) exposed to chilling, MeJA decreased Put accumulation, whereas Spd and Spm accumulation increased [92]. In mangoes and apples, higher free Spd and Spm content correlated with low-temperature stress tolerance [125,126]. In rice treated with MeJA before chilling, a significant increase in free Put and Spm accumulation in the shoots and roots was observed. In contrast, the free Spd content was reduced. Further analyses have revealed that in rice seedlings, accumulation of Put induced by MaJA is the result of the activation of the ADC pathway, but not the ODC pathway [32]. In *Indica-japonica* hybrid rice, exogenous Spd enhanced CK and GA3 content during chilling, indicating a possible crosstalk between PAs and these plant hormones [93].

## 4. Conclusions and Future Perspectives

Permanent changes in environmental conditions challenge equitable agriculture and efficient plant production. The increasing mean air temperature and the accompanying drought, as well as soil salinity, are environmental factors that are subject to rapid changes in the coming years. The unfavorable direction of these changes affects plant growth and development. Therefore, one of the priorities of the scientific world is to acquire and enhance knowledge about the mechanisms that allow plants to deal with abiotic stresses and to develop strategies to introduce transgenic plants to agriculture. Exploration of PAs’ mode of action in plant tolerance of abiotic stress seems to be a crucial step in this process. An important aspect of PAs’ action in the response to abiotic stress is their interactions with plant hormones.

This review presents the relationship between PAs and plant hormones in plant tolerance of abiotic stress (Table 2). Our study has shown the favorable effect of the co-application of exogenous PAs and phytohormones, such as CKs and BRs, on stressed plants. We have demonstrated that PAs affect the accumulation of plant hormones and the expression of their biosynthesis genes during abiotic stress. This study is a good starting point for further research focusing on the regulation of PAs and plant hormones metabolism at the post-transcriptional and post-translational levels in plants under abiotic stress. Furthermore, it is worth investigating the possible function of PAs in the regulation of plant hormone signaling during abiotic stress. Several components, such as Ethylene Response Sensor 1 (ERS1), Ethylene Insensitive 2 (EIN2), EIN3-like (EIL), and Ethylene Responsive Factors (ERF), participate in ETH signaling and play a relevant role in plant response to salt stress [127,128,129]. PAs regulate some components of the ETH signaling pathway [63]. It would be interesting to explore the connections between PAs and plant hormones—such as ETH, ABA, GAs, and JAs signaling pathways during abiotic stress—and establish the role of those relationships in inducing plant tolerance of adverse environmental conditions.

In addition, it seems interesting to examine the role of NO in this aspect. Similar to PAs, NO interacts with plant hormones during plant responses to abiotic stress [130]. Therefore, it would be interesting to investigate the possible mechanisms involving PAs, NO, and plant hormones that induce plant tolerance to stress. A crucial step in achieving this goal is to establish the role of PAs in NO production.

## Figures and Tables

**Table 1:** Representative evidence for the interaction between PAs and plant hormones during plant growth and development. 6-BA, 6-benzylaminopurine; IAA, indole acetic acid; iP, isopentenyl adenine; NaSA, sodium salicylate.

PAs-Plant Hormone Crosstalk	Plant Species	Exogenous Treatment, Gene Mutation or Over-Expression	Effect (or Outcome)	References
ABA	Maize	Exogenous ABA	Increased PAO activity, enhanced *PAO* expression	[52]
	Tomato	Exogenous ABA	Induced expression of *SlPAO2-4* genes, reduced expression *SlPAO1* gene and *SlPAO6-7* genes	[44]
	Cucumber	Exogenous ABA	Up-regulated expression of *CsSAMS1* and *CsSAMS2* in leaves, increased expression of *CsSAMS1* and inhibited expression of *CsSAMS2* in roots	[53]
	Rice	Exogenous ABA	Enhanced expression of *OsPAO1*	[54]
	*Arabidopsis*	Exogenous ABA	Up-regulated expression of *ADC2*, *ARGAH1*, *ARGAH2*, *AIH*, *CPA*, *SPDS2*, *SPMS*, *SAMDC1-2*, *PAO1-4*, *CuAOγ1/CuAO1*, *CuAOδ*	[45]
	*Arabidopsis*	Exogenous Spd and exogenous Spm	Increased ABA content, up-regulated *NCED3* expression	[55]
Auxin	Tomato	Exogenous IAA	Stimulated expression of *SlPAO2*, *SlPAO4* and *SlPAO7* genes, reduced expression of *SlPAO6*	[44]
	*Arabidopsis*	Exogenous IAA	Up-regulated expression of *ADC2*, *ACL5*, *SAMDC4/BUD2*, *PAO1*, *PAO2*, *PAO5*, reduced expression of *CuAOα2* and *CuAOα3/CuAO2*	[45]
	*Arabidopsis*	*acl5-1*	Up-regulation of genes related to auxin biosynthesis (*YUCCA2*), methylation (*IAMT1*), transport (*PIN-FORMED1* and *PIN6*) and signal transduction (*MP/ARF5*)	[49]
BRs	Tomato	Exogenous EBL	Modulated PAs content in plant organs	[56]
CKs	Cucumber	Exogenous KN	Increased Put content, decreased Spd content, reduced SAMDC activity and stimulated PAO activity	[57]
	Tomato	Exogenous 6-BA	Stimulated expression of *SlPAO2-5* genes and reduced expression of *SlPAO6-7* genes	[44]
	Rice	Exogenous iP	Up-regulated expression of *OsPAO1*, *OsPAO3*, *OsPAO5* and *OsPAO7*	[54]
	*Arabidopsis*	*bud2*	Enhanced CKs production, hypersensitivity to exogenous CKs	[58]
ETH	Tomato	Exogenous ethephon	Stimulated expression of *SlPAO1-2* genes and *SlPAO4* gene, decreased expression of *SlPAO5-7* genes	[44]
	*Arabidopsis*	Exogenous ACC	Enhanced expression of *ADC2* and reduced expression of *CuAOα2*	[45]
	Peach	Exogenous Spd	Decreased ETH content, enhanced expression of *ETR1* and *ERS1* in fruits	[48]
	Olive (ARB cv.)	Exogenous Put	Negatively regulated expression of *OeACO2* and *OeCTR1*, and positively regulated *OeERS1* expression in fruits	[59]
	Olive (ARB cv.)	Exogenous Spd	Up-regulated expression of *OeERS1*, down-regulated expression of *OeCTR1* and *OeEIL2* in fruits	[59]
	Grapes	Exogenous guazatine	Put accumulation, stimulated expression of *EIN3* and *EBF2* genes	[60]
	tomato	*Nr*	Enzymes of Put production responded to exogenous SA in a light- and ET-dependent manner	[46]
GAs	Pea	Exogenous GA3	Increased *ADC* expression, decreased *ODC* expression	[61]
	Grape	Exogenous GA3	Enhanced free Put content	[62]
	Tomato	Exogenous GA3	Up-regulated expression of *SlPAO2-3* genes and *SlPAO5* gene	[44]
	Rice	Exogenous GA	Up-regulated expression of *OsPAO5* and *OsPAO7*	[54]
	*Arabidopsis*	*ADC2*	Lower GA1, GA4 and GA9 content, reduced expression of genes related to GA biosynthesis (*AtGA20ox1*, *AtGA3ox1* and *AtGA3ox3*)	[47]
	Tomato	*ySAMdc*	Enhanced expression of genes encoding GA 2-oxidase and GA 20-oxidase during fruit ripening	[63]
	*Arabidopsis*	Exogenous Put	Up-regulation *GA3ox1* expression	[55]
	*Arabidopsis*	Exogenous Spm	Reduced GA1 content, down-regulation *GA3ox1* expression	[55]
JAs	Tomato	Exogenous MeJA	Enhanced expression of *SlPAO1-2* genes and decreased expression of *SlPAO4* and *SlPAO6-7* genes	[44]
	Cucumber	Exogenous MeJA	Modulated expression of *CsSAMS1* and *CsSAMS2* genes in roots and leaves during treatment	[53]
	Rice	Exogenous JA	Up-regulated expression of *OsPAO1-3* and *OsPAO6-7*	[54]
	*Arabidopsis*	Exogenous MeJA	Enhanced expression of *ARGAH1*, *ARGAH2*, *PAO3* and *CuAOα3/CuAO2*	[45]
	*Arabidopsis*	Exogenous Spm	Up-regulation of JA biosynthesis genes (*LOX1-4*, *AOS*, *AOC1*, *AOC2*, *OPR3*, *OPCL1*, *CYP94B3*) and JA signaling genes (*JAZ1*, *JAZ5*, *JAZ6*, *JAZ7* and *JAZ10*)	[64]
	*Arabidopsis*	Exogenous tSpm	Up-regulation of JA biosynthesis genes (*LOX1-4*, *AOS*, *AOC1*, *AOC2*, *OPR3*, *OPCL1*, *CYP94B3*), JA signaling genes (*JAZ1*, *JAZ5*, *JAZ6*, *JAZ7*, *JAZ10* and *MYC4*) and JA response marker genes (*VSP2* and *PDF1.2*)	[64]
	*Arabidopsis*	Exogenous Spd	Lower JA content, reduced *AOS* expression	[55]
	*Arabidopsis*	Exogenous Spm	Lower JA content, enhanced *AOS* expression	[55]
SA	Tomato	Exogenous SA	Stimulated expression of *SlPAO2-4* genes and reduced expression of *SlPAO6-7* genes	[44]
	Cucumber	Exogenous SA	Enhanced expression of *CsSAMS1* and *CsSAMS2* in leaves	[53]
	*Arabidopsis*	Exogenous SA	SA modulated PAs content and PA metabolism gene expression	[65]
	*Arabidopsis*	*mpk6-2*	Decreased Put content	[65]
	*Arabidopsis*	Exogenous NaSA	Up-regulated expression of *ADC2*, *AIH*, *SPMS* and *PAO1*, down-regulated expression of *CuAOα2*, *CuAOα3/CuAO2* and *CuAOγ2*	[45]
	*Arabidopsis*	Exogenous PAs	Modulation of PAs biosynthesis and catabolism gene expression in *eds5* and *sid2* mutants	[55]

**Table 2:** Summary of evidence for the interaction between PAs and plant hormones in response to abiotic stresses.

Abiotic Stress	PAs-Plant Hormone Crosstalk	Plant Species	Exogenous Treatment, Gene Mutation or Over-Expression	Effect (or Outcome)	References
Drought, osmotic stress	ABA ↔ PAs	*Vicia faba*	Exogenous PAs	Induced stomatal closure	[72]
	ABA ↔ PAs	*Vicia faba*	Exogenous ABA	Higher CuAO and NADPH oxidase activity leading to enhanced H_2_O_2_ production	[73]
	ABA ↔ PAs	*Arabidopsis*	Exogenous ABA	Higher expression of *AtPAO2* in guard cells	[74]
	ABA ↔ PAs	*Arabidopsis*	Exogenous PAs	Induced stomatal closure, enhanced NO and ROS levels in guard cells due to NADPH oxidase and AO activity	[75]
	ABA ↔ PAs	*Arabidopsis*	*aba2*, *abi1*	No observed dehydration-inducible expression of *ADC2*, *SPDS1* and *SPMS*	[76]
	ABA ↔ PAs	*Arabidopsis*	-	Presence of ABREs in promoter regions of *ADC2*, *SPDS1* and *SPMS* genes	[77]
	ABA ↔ PAs	*Arabidopsis*	-	Enhanced expression of ABA-inducible and drought-responsive genes (*RD29A* and *RD22*) and PAs biosynthesis genes (*ADC1*, *ADC2*, *SPDS1*, *SPDS2*, *SPMS* and *SAMDC1*)	[68]
	ABA ↔ PAs	Rice	-	Presence of putative cis-acting elements, such as ABRE, LTRE, MYB and W-box, in the promoter region of *SAMDC* gene	[78]
	ABA ↔ PAs	Rice	*OsHSFA3*	Higher ABA level, increased PAs content, up-regulation of *ADC1*, *ADC2*, *SPDS1* and *SPMS* expression	[79]
	ABA ↔ PAs	*Lotus tenuis*	*ADC*	Increased Put content, expression of *NCED* gene and ABA accumulation	[80]
	ABA ↔ PAs	Wheat	Exogenous Put	Enhanced expression of *NCED*	[81]
	ABA ↔ PAs	Wheat (drought-susceptible cv.)	Exogenous Spm	Further escalation in ABA level	[82]
	ABA ↔ PAs	Wheat (drought-resistant cv.)	Exogenous Spd	Reduced ABA level	[82]
	ABA ↔ PAs	Wheat	Exogenous ABA	Enhanced expression of *ADC*, decreased expression of *SPDS* and *PAO*	[81]
	ETH ↔ PAs	Wheat	Exogenous Spd and exogenous Spm	Reduction of ETH evolution rate, increased CKs and ABA level	[83]
	ETH ↔ PAs	Wheat	Exogenous Put	Increased ETH evolution rate, excessive ABA accumulation	[83]
	JAs ↔ PAs	Rice	Exogenous MeJa	Negatively regulation of *OsAdc*, *OsSamdc* and *OsSpds* genes	[84]
	JAs ↔ PAs	Maize	Exogenous Spd	Decreased MeJA content	[39]
	CKs ↔ PAs	Maize	Exogenous Spd	Increased ZR content	[39]
	GAs ↔ PAs	Wheat	Exogenous Spd and exogenous Spm	Higher GA (GA1 + GA4) content during seed germination	[83]
	GAs ↔ PAs	Bentgrass	Exogenous Spm	Increased GA1 content	[85]
	GAs ↔ PAs	Bentgrass	Exogenous Spd	Increased GA4 and GA20 content	[3,85]
Salt stress	ABA ↔ PAs	*Arabidopsis*	*SAMDC1*	Improved expression of ABA-induced genes (*NCED* and *RD29A*)	[21]
	ABA ↔ PAs	*Arabidopsis*	*spms*, *acl5/spms*	Decreased *NCED3* expression	[21]
	ABA ↔ PAs	*Arabidopsis*	*atpao5-3*	Higher ABA content, up-regulated expression of *NCED* and *RD29B*	[64]
	BRs ↔ PAs	Tomato	Exogenous EBL	Increased PAs level in the middle leaf and fruits, higher Spm level in the stem higher (Spd + Spm)/Put ratio in the fruits	[56]
	BRs ↔ PAs	Lettuce	Exogenous DI-31	Enhanced PAs accumulation in shoots, reduced Spd and Spm content in roots	[86]
	BRs ↔ PAs	Canola	Exogenous EBL	Increased accumulation of Put in cotyledons and reduced in hypocotyls and radicles, reduced H_2_O_2_ content, decreased DAO activity and increased PAO activity	[14]
	ETH ↔ PAs	Rice	Exogenous Put	Enhanced ETH production	[67]
	ETH ↔ PAs	Tobacco	*AS-ZmPAO*	Up-regulation of *ACCSyn* and *ACCOx* gene expression	[4]
	ETH ↔ PAs	Tomato	*Nr*	Lower Spm content, enhanced DAO, and PAO activity	[46]
	JAs ↔ PAs	Cucumber	Exogenous Spd	Decreased JA content	[87]
	JAs ↔ PAs	*Arabidopsis*	*atpao5-3*	Increased JA accumulation	[64]
	CKs ↔ PAs	*Vigna sinensis*	Exogenous KN	Increased PAs content	[88]
	GAs ↔ PAs	Cucumber	Exogenous Spd	Enhanced *GT-3b* expression, increased GA3 level	[33]
Heavy metal stress	BRs ↔ PAs	Radish	Exogenous EBL	Increased Put content, decreased Spd content	[56]
	BRs ↔ PAs	Mustard	Exogenous EBL	Increased PAs level in leaves	[89]
	BRs ↔ PAs	Mustard	Exogenous EBL and SA	Increased Spd level in roots	[90]
	ETH ↔ PAs	Wheat	Exogenous Put	Improved root elongation, decreased ACS activity and ETH level	[6]
	ETH ↔ PAs	Cucumber	Exogenous 2-OHMT	Higher PAs content, reduction of PAO activity, increased *CS-ERS* expression	[7]
Low-temperature stress	ABA ↔ PAs	Tomato	Exogenous Put	Increased ABA level and *LeNCED1* expression, role of ABA in Put-induced tolerance	[35]
	ABA ↔ PAs	*Arabidopsis*	*adc*	Reduced ABA accumulation, lower *NCED* expression, down-regulation of ABA-regulated genes	[12]
	ABA ↔ PAs	*Arabidopsis*	*adc*	Reduced ABA level, decreased ABA-dependent gene induction, decreased freezing tolerance	[91]
	JAs ↔ PAs	Zucchini	Exogenous MeJA	Higher Put content, lower Spd and Spm content	[92]
	JAs ↔ PAs	Rice	Exogenous MeJA	Shoots: increased Put and Spm content, reduced Spd content, increased ADC activity, reduced ODC and SAMDC activity; roots: increased Put and reduced Spd content, increased ADC and SAMDC activity, reduced ODC activity	[32]
	CKs ↔ PAs	*Indica-japonica* hybrid rice	Exogenous Spd	Higher ZR content	[93]
	GAs ↔ PAs	*Indica-japonica* hybrid rice	Exogenous Spd	Higher GA3 content	[93]

## Data Availability

Not applicable.
